# Hand-Book for the Military Surgeon

**Published:** 1861-06

**Authors:** 


Hand-Book for the Military Surgeon:—Being a Compendium of the Duties of the Medical Officer in the Field, the Sanitary Management of the Camp, the Preparation of Food, etc.; with Forms for the Requisitions for Supplies, Returns, etc.; Diagnosis and Treatment of Camp Dysentery; and all the Important Points in *War Surgery:* Including Gun-Shot Wounds, Amputations, Wounds in the Chest, Abdomen, Arteries and Head, and the Use of Chloroform. By Charles S. Tripler, A. M., M. D., Surgeon United States Army. And George C. Blackman, M. D., F. R. M. S., Professor of Surgery in the Medical College of Ohio, Surgeon to the Commercial Hospital, St. John's Hospital, etc. Cincinnati: Robert Clark & Co., Publishers. 1861.

The first, of numbers to be looked for in these days of the reign of *horrida Bella*, and of utter stagnation among the book publishers, in aught not directly appertaining to the tented field. In fact, this and its companion, elsewhere noticed, both confess to be solely the result of the energy of "the enterprising publishers," and not the mature outgrowth of their respective authors. Not, however, that this work is not valuable and eminently opportune in its appearance; for the position of Dr. Tripler, U. S. A., and within a very few removes of the oldest surgeon in the army, is sufficient guarantee of actual worth, aside from the high endorsement of Prof. Blackman's name and active co-operation.

The substance of the work is an extended and revised form of a series of lectures on Military Surgery, which Dr. Tripler has very acceptably delivered in the Medical College of Ohio, during the past three years. These lectures cover the ground of *Duties of the Medical Officer—organization of field-hospitals—mode of procuring supplies—military hygiene—preservation of health of troops in campaign—camp dysentery—general history, character and treatment of gun-shot wounds, amputations, and wounds of the chest.* Prof. Blackman supplies the chapters on *wounds of the abdomen, head and arteries;* and the chapter on the *Use of Chloroform* is from Mr. Macleod's valuable work on the Crimean War.

